# Spatiotemporal dynamics in human visual cortex rapidly encode the emotional content of faces

**DOI:** 10.1002/hbm.24226

**Published:** 2018-06-08

**Authors:** Diana C. Dima, Gavin Perry, Eirini Messaritaki, Jiaxiang Zhang, Krish D. Singh

**Affiliations:** ^1^ BRAIN Unit, School of Medicine Cardiff University Cardiff CF24 4HQ United Kingdom; ^2^ Cardiff University Brain Research Imaging Centre (CUBRIC), School of Psychology, Cardiff University Cardiff CF24 4HQ United Kingdom

**Keywords:** face perception, magnetoencephalography (MEG), multivariate pattern analysis (MVPA), threat bias

## Abstract

Recognizing emotion in faces is important in human interaction and survival, yet existing studies do not paint a consistent picture of the neural representation supporting this task. To address this, we collected magnetoencephalography (MEG) data while participants passively viewed happy, angry and neutral faces. Using time‐resolved decoding of sensor‐level data, we show that responses to angry faces can be discriminated from happy and neutral faces as early as 90 ms after stimulus onset and only 10 ms later than faces can be discriminated from scrambled stimuli, even in the absence of differences in evoked responses. Time‐resolved relevance patterns in source space track expression‐related information from the visual cortex (100 ms) to higher‐level temporal and frontal areas (200–500 ms). Together, our results point to a system optimised for rapid processing of emotional faces and preferentially tuned to threat, consistent with the important evolutionary role that such a system must have played in the development of human social interactions.

## 1 INTRODUCTION

Owing to their high behavioral relevance, emotional cues present in facial expressions are rapidly processed, with a vast literature pointing to the rapid identification of threat‐related expressions (Fox et al., 2000; Öhman, Lundqvist, & Esteves, 2001; Pichon, De Gelder, & Grèzes, 2012). According to the classic model of face perception (Haxby et al., 2000), information about facial expression is represented in the superior temporal sulcus (STS) based on inputs from the occipital face area (OFA), while the fusiform face area (FFA) extracts invariant features such as face identity. This model has been challenged by evidence of expression processing in the FFA (Bernstein & Yovel, 2015) and of parallel pathways linking the visual cortex and the core face‐selective areas (Pyles et al., 2013), suggested that information is extracted from faces by distributed and interacting modules (Duchaine & Yovel, 2015).

Rapid face processing facilitates fast evaluation and top‐down modulation by higher‐level areas (Adolphs, 2002). However, there is some debate on how such rapid perception is accomplished. A fast subcortical thalamus‐amygdala route bypassing the visual cortex is thought to transmit coarse face‐related information (LeDoux, 2009; Morris et al., 1998), but its role in face perception is controversial (Krolak‐Salmon et al., 2004; Pessoa & Adolphs, 2011), including whether it is fear‐specific (Méndez‐Bértolo et al., 2016) or non‐specific to expression (Garvert, Friston, Dolan, & Garrido, 2014; McFadyen et al., 2017). On the other hand, multiple fast cortical pathways forming part of a feedforward and feedback mechanism consistute an equally plausible mechanism for rapid expression perception (Liu & Ioannides, 2010; Pessoa & Adolphs, 2010).

Furthermore, electrophysiological investigations of emotional face processing in humans are not always in agreement on the temporal dynamics of expression perception. Early emotional modulations of the posterior P1 evoked response component (∼100 ms) are sometimes reported (Aguado et al., 2012; Eger, Jedynak, Iwaki, & Skrandies, 2003; Halgren et al., 2000; Pourtois et al., 2005), with other studies failing to find early effects (Balconi, & Pozzolili, 2003; Frühholz, Jellinghaus, & Herrmann, 2011; Krolak‐Salmon, Fischer, Vighetto, & Mauguiere, 2001; Schupp et al., 2004). On the other hand, modulations of the N170 face‐responsive component (120–200 ms) are consistently reported (see Hinojosa, Mercado, & Carretiéé, 2015 for a meta‐analysis).

While these results point to relatively late effects, high‐level categorization has been shown to occur at shorter latencies in the visual system of primates, especially when tested using sensitive multivariate methods (Cauchoix et al., 2016). In humans, multivariate pattern analysis (MVPA) of non‐invasive electrophysiological data has shown potential to achieve a similar level of sensitivity, demonstrating rapid categorization along the ventral stream (Cauchoix, Barragan‐Jason, Serre, & Barbeau, 2014; Isik, Meyers, Leibo, & Poggio, 2014; Ramkumar, Hansen, Pannasch, & Loschky, 2016). Fast decoding of object category was achieved at ∼100 ms from small neuronal populations in primates (Hung & Poggio, 2005) and from invasively recorded responses in human visual cortex (Li & Lu, 2009). Furthermore, recent applications of MVPA to electrophysiological data have resolved face identity processing to early latencies (50–70 ms after stimulus onset; Davidesco et al., 2014; Nemrodov et al., 2016; Vida, Nestor, Plaut, & Behrmann, 2017). In addition to revealing the temporal dynamics of visual processing, multivariate methods have furthered our understanding of the transformations performed by cells in macaque face patches to encode face identity (Chang & Tsao, 2017) and have allowed face reconstruction based on non‐invasive neural data in humans (Nemrodov et al., 2018; Nestor, Plaut, & Behrmann, 2016).

Previous studies have demonstrated successful decoding of facial expression from face‐selective areas using fMRI (Wegrzyn et al., 2015; Zhang et al., 2016a). Some studies have also decoded emotion from EEG data (Kashihara, 2014; Li et al., 2009; Petrantonakis, & Hadjileontiadis, 2010) and target happy expressions from MEG data (Cecotti et al., 2017), while an intracranial EEG study has demonstrated late decoding of facial expression (fear and happiness) from the human fusiform gyrus (Tsuchiya et al., 2008). However, MVPA has not so far been used, to our knowledge, to study the spatiotemporal dynamics of expression processing. In the present study, we exploited the temporal and spatial resolution of MEG and the ability of MVPA to identify differences in activation patterns as a window into the whole‐brain dynamics of emotional face perception.

MEG can resolve the timecourse of fast processes with a resolution in the order of milliseconds, while source localization and information‐based mapping through multivariate techniques can effectively increase its spatial resolution (Cichy et al., 2015; Kriegeskorte, Goebel, & Bandettini, 2006). MVPA offers increased sensitivity through its ability to extract information from responses at multiple locations in space and time, and it can thus resolve differences in overlapping patterns that averaging‐based statistical analyses fail to detect (Norman, Polyn, Detre, & Haxby, 2006). At the minimum, successful decoding points to the availability of information at certain time points and sources, although it does not tell us how or if this information is used in neural computation (DeWit et al., 2016; Kriegeskorte et al., 2006). We thus focus on MVPA in the present study as a tool that can elucidate previous mixed results on the temporal dynamics of expression processing by examining pattern information that may not be present in averaged evoked responses.

Using MVPA, we first interrogated the fine temporal dynamics underpinning expression perception, including discrimination between emotional and neutral expressions and between different emotions. Next, we applied a novel approach to source‐space decoding to track the brain regions encoding the emotional content of faces and their relative contribution over time. We were thus able to identify early differences between responses to angry faces and happy/neutral faces within 100 ms of stimulus onset and we localized them to the visual cortex, while later responses originated in higher‐level temporal and frontal cortices. Our results suggest that the perceptual bias towards threatening expressions begins with the early stage of visual processing, despite a lack of significant differences in trial‐averaged ERFs.

## 2 MATERIALS AND METHODS

### 2.1 Participants

The participants were 15 healthy volunteers (8 females, mean age 28, *SD* 7.63) with normal or corrected‐to‐normal vision. All volunteers gave informed written consent to participate in the study in accordance with The Code of Ethics of the World Medical Association (Declaration of Helsinki). All procedures were approved by the ethics committee of the School of Psychology, Cardiff University.

### 2.2 Stimuli

Forty‐five angry, happy and neutral male and female face images (15 images per condition) were selected from the NimStim database (Tottenham et al., 2009). We note that the NimStim database includes both closed‐mouth (low arousal) and open‐mouth (high arousal) versions of each emotional expression; for this study, we selected closed‐mouth neutral expressions, open‐mouth happy expressions, and a balanced set of closed‐mouth and open‐mouth angry expressions, which accounted for the higher arousal associated with negative expressions. In practice, this stimulus selection enhances visual differences (i.e., in terms of visible teeth) between the happy and neutral face sets.

**Figure 1:**
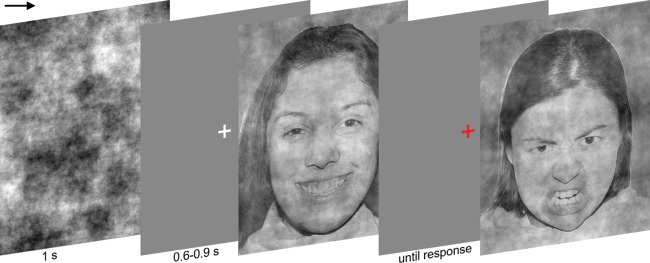
Experimental paradigm, together with examples of one scrambled image and two face stimuli from the NimStim database, after normalization of Fourier amplitudes [Color figure can be viewed at http://wileyonlinelibrary.com]

The fourth condition contained 15 scrambled face stimuli, which were noise images created by combining the average Fourier amplitudes across stimuli with random phase values. The images were 506 × 560 pixels in size and were converted to grayscale (Figure 1). To ensure matched low‐level properties between stimuli, the 2D Fourier amplitude spectrum of each image was set to the average across all stimuli. This was done by calculating the average amplitude spectrum across images in the Fourier domain, and replacing individual amplitude spectra with the average when performing the inverse transformation of each image.

### 2.3 Data acquisition

All participants underwent a whole‐head T1‐weighted MRI scan on a General Electric 3 T MRI scanner using a 3D Fast Spoiled Gradient‐Recalled‐Echo (FSPGR) pulse sequence in an oblique‐axial orientation with 1 mm isotropic voxel resolution and a field of view of 256 × 192 × 176 mm (TR/TE = 7.9/3.0 ms, inversion time = 450 ms, flip angle = 20°).

Whole‐head MEG recordings were made using a 275‐channel CTF radial gradiometer system at a sampling rate of 600 Hz and an associated anti‐aliasing low‐pass filter at 150 Hz. Three of the sensors were turned off due to excessive sensor noise and an additional 29 reference channels were recorded for noise rejection purposes. The data were collected in 2.5 s epochs centered around the stimulus onset. A continuous bipolar electrooculogram (EOG) was recorded to aid in offline artefact rejection.

Stimuli were centrally presented on a gamma‐corrected Mitsubishi Diamond Pro 2070 CRT monitor with a refresh rate of 100 Hz and a screen resolution of 1024 × 768 pixels. Participants viewed the stimuli from a distance of 2.1 m at a visual angle of 8.3° × 6.1°.

Participants underwent two scanning sessions with a few minutes of break in between. Each session comprised 360 trials, with the 15 images corresponding to each condition presented six times in random order. On each trial, the stimulus was presented on a mean gray background for 1 s, followed by an interstimulus interval with a duration selected at random from a uniform distribution between 600 and 900 ms (Figure 1). A white fixation cross was presented at the center of the screen throughout the experiment. Participants performed a change detection task to ensure maintained attention: the fixation cross turned red at the start of a pseudorandom 10% of trials (during the inter‐stimulus interval) and participants had to press a button using their right index finger in order to continue. The paradigm was implemented using Matlab (The Mathworks, Natick, MA) and the Psychophysics Toolbox (Brainard, 1997; Kleiner et al., 2007; Pelli, 1997).

Participants were seated upright while viewing the stimuli and electromagnetic coils were attached to the nasion and pre‐auricular points on the scalp to determine head location. Head position was monitored continuously and head motion did not exceed 6.6 mm in any given session. High‐resolution digital photographs were used to verify the locations of the fiducial coils and co‐register them with the participants' structural MRI scans.

### 2.4 Data analysis

#### 2.4.1 Pre‐processing

Prior to sensor‐space analyses, the data were pre‐processed using Matlab and the FieldTrip toolbox (Oostenveld, Fries, Maris, & Schoffelen, 2011; http://www.fieldtriptoolbox.org). Trials containing eye movement or muscle artefacts were rejected after visual inspection. One participant was excluded due to excessive artefacts and analysis was performed on the remaining 14 subjects. Across the remaining subjects, the percentage of trials excluded did not exceed 12.7% (mean 40 trials excluded across both sessions, *SD* 24.3), and the number of trials excluded did not significantly differ between conditions (*p *=* *.86, *F*(2.2,28.9) = 0.18).

To monitor head motion, the position of the three fiducial coils relative to a fixed coordinate system on the dewar was continuously recorded during data acquisition. Head motion was quantified as the maximum displacement (difference in position between sample points) of the three coils during any given trial. Using this metric, we excluded trials with maximum motion of any individual coil in excess of 5 mm. To account for changes in head position, head coil position was changed to the average position across trials for each dataset.

For sensor‐space analyses, a 50 Hz comb filter was used to remove the mains noise and its harmonics and baseline correction was applied using a time window of 500 ms prior to stimulus onset.

#### 2.4.2 Event‐related field (ERF) analysis

We inspected event‐related fields in order to examine differences between conditions present in single‐channel responses. The data were bandpass‐filtered between 0.5 and 30 Hz using fourth‐order IIR Butterworth filters. ERFs were realigned to a common sensor position (Knösche, 2002) and averaged across subjects (Supporting Information Figure S1a). We then identified three time windows of interest based on local minima in the global field power across all face conditions (Supporting Information Figure 1b; Perry & Singh, 2014): ∼60–127 ms (M100), 127–173 ms (M170), and 173–317 ms (M220). ERF responses were averaged within each time window of interest. For each time window, we tested for differences between trial‐averaged responses to neutral and scrambled faces and between emotional faces using a paired *t* test and a repeated‐measures ANOVA respectively and randomization testing (5,000 iterations, corrected using the maximal statistic distribution across sensors).

#### 2.4.3 MVPA pre‐processing and feature selection

***Sensor space*.** Prior to sensor‐space MVPA analyses, the data were averaged in groups of 5 trials to improve SNR (Grootswagers et al., 2017; Isik et al., 2014; Wardle et al., 2016). The number of observations was not significantly different between conditions (Angry: 33.6 ± 1.6; Happy: 33.4 ± 1.4; Neutral: 33.5 ± 1.1; Scrambled: 33.6 ± 1; *F*(3,13) = 0.64, *p *=* *.59). To assess differences between responses to neutral and emotional faces as well as between different emotional expressions, binary classification was applied to all pairs of emotional conditions.

We assessed the presence, latency and coarse spatial location of expression‐specific information at the sensor level by performing within‐subject time‐resolved classification on data from four anatomically defined sensor sets (occipital, temporal, parietal, and frontocentral; Figure 5a). MVPA was performed at each sampled time point (every ∼1.67 ms) between 0.5 s pre‐stimulus onset and 1 s post‐stimulus onset. Compared to a whole‐brain approach, this method served to reduce the number of features while also providing some spatial information.

To maximize the number of informative features used as input to the classifier, we conducted an additional sensor‐space MVPA analysis in which feature selection was performed based on differences between faces and scrambled stimuli. This ensured unbiased feature selection based on an orthogonal contrast and led to the selection of sensors responding most strongly to faces, in order to maximize the interpretability of our results.

To determine sensors responding differentially to faces and scrambled stimuli we used a searchlight MVPA approach, whereby each MEG channel and its neighboring sensors, defined according to a Fieldtrip template based on the CTF 275‐sensor array configuration, were entered separately into the MVPA analysis. Searchlights were defined to include only sensors directly connected to the centroid according to the template, and searchlight size thus ranged between 4 and 10 sensors (mean 7.36, *SD* 1.12). The analysis was performed using time windows of ∼16 ms (10 sampled time points) and stratified five‐fold cross‐validation was used to evaluate classification performance. Data from the cluster centroids found to achieve above‐chance decoding performance in 100% of participants (regardless of latency) were then entered into the three emotional expression classification analyses (Figure 5b).

Two additional feature selection methods based on the face versus scrambled contrast, which achieved lower or similar decoding performance, are described in the supporting information (Supporting Information Analysis S1).

***Source space*.** To move beyond the limitations of sensor‐space spatial inference in our MVPA analysis (including concerns of signal leakage, head motion and inter‐individual variability; Zhang et al., 2016), the data were projected into source space using the linearly constrained minimum variance (LCMV) beamformer (Hillebrand et al., 2005; Van Veen, van Drongelen, Yuchtman, & Suzuki, 1997). This approach combines the forward model and the data covariance matrix to construct an adaptive spatial filter. Beamformer weights were normalized by their vector norm to alleviate the depth bias of MEG source reconstruction (Hillebrand et al., 2012). The participant's MRI was used to define the source space with an isotropic resolution of 6 mm and the output for each location was independently derived as a weighted sum of all MEG sensor signals using the optimal source orientation (Sekihara, Nagarajan, Poeppel, & Marantz, 2004).

The data were projected into source space using trials from all conditions filtered between 0.1 and 100 Hz to calculate the beamformer weights. A frequency analysis was performed using the multitaper method based on Hanning tapers in order to identify the peak virtual channel in each of 84 Automated Anatomical Labeling (AAL; Tzourio‐Mazoyer et al., 2002) atlas‐based ROIs (excluding the cerebellum and some deep structures; see Figure 6a). The classifier input consisted of the raw time‐series for each of the 84 virtual sensors, baseline corrected and averaged in groups of 5 trials to improve SNR. Decoding was performed per sampled time point as in sensor space.

#### 2.4.4 Classifier training and testing

A linear L1 soft‐margin Support Vector Machine (SVM) classifier (Burges, 1997) was implemented in Matlab using the Machine Learning and Statistics Toolbox and the Bioinformatics Toolbox (Mathworks, Inc.). SVM finds the optimal separating hyperplane between classes and implements a sparse solution by assigning non‐zero weights exclusively to data points situated closest to the decision boundary (support vectors). It is known to generalize well even in cases of high dimensionality due to its in‐built regularization (Nilsson, Pena, Bjorkegren, & Tegner, 2006).

Stratified five‐fold cross‐validation was implemented for training and testing and data points were standardized for each time window using the mean and standard deviation of the training set. The box constraint parameter *c*, which controls the maximum penalty imposed on margin‐violating observations, was set to 1.

#### 2.4.5 Computing relevance patterns in source space

For each decoding problem, participant and time point, the SVM model based on source‐space data was retrained on the full dataset to obtain the final model and calculate the weight vector. The weight vector for a linear SVM is based on the Lagrange multipliers assigned to each data point. To achieve interpretable spatial patterns (Haynes, 2015), feature weights were transformed into relevance patterns through multiplication by the data covariance matrix (Haufe et al., 2014). This allowed us to dynamically and directly assess the relative importance of all virtual electrodes used in source‐space decoding, as each ROI was represented by one feature and each decoding iteration was run on the whole brain.

#### 2.4.6 Significance testing

To quantify classifier performance, we report average accuracies across subjects (proportions of correctly classified cases), as well as F1 scores (harmonic means of precision and sensitivity) and bias‐corrected and accelerated bootstrap confidence intervals using 1,000 resampling iterations (Efron, 1987; Efron & Tibshirani, 1986).

In order to account for the potentially skewed distribution of cross‐validated accuracies given our limited observation numbers (Jamalabadi et al., 2016), significance was assessed using permutation testing, which offers a robust measure of significance compared to theoretical chance levels (Combrisson & Jerbi, 2015) or binomial tests (Noirhomme et al., 2014). For each individual dataset, labels were shuffled 1,000 times across the training and test sets to create an empirical null distribution and classification was performed on the randomized data at the time point achieving the highest classification performance across subjects on the real data. For searchlight classification, *p* values were calculated for each subject and combined to achieve a group map quantifying the proportion of subjects achieving significance in each searchlight (Pereira, & Botvinickck, 2011). For all other analyses, randomization was performed within‐subject and empirical null distributions were calculated in an identical manner as the observed statistic (i.e., average accuracy over subjects).

To correct for multiple comparisons, we tested average accuracies against the omnibus null hypothesis by thresholding using the maximum accuracy distribution (Nichols & Holmes, 2002; Singh, Barnes, & Hillebrand, 2003). For classification on different sensor sets, this was done by selecting the maximum average performance across sensor sets to create a null empirical distribution. For searchlight classification, *p* values were thresholded using the maximum performance across sensor clusters. For sensor‐space classification based on feature selection and for source‐space classification, *p* values were adjusted using the false discovery rate and cluster‐corrected across time. Permutation *p* values were calculated taking the observed statistic into account, using the conservative estimate p=(b+1)/(m+1), where *b* is the number of simulated statistics greater than or equal to the observed statistic and *m* is the number of simulations (Phipson, & Smythth, 2010).

To identify the ROIs significantly contributing to decoding performance in source space, permutation testing (5,000 sign‐flipping iterations) was applied to baselined mean relevance patterns for each ROI and time window. P‐values were corrected for multiple comparisons using the maximum statistic distribution across ROIs, and a further Bonferroni correction was applied to account for the multiple time windows tested.

Decoding was also performed on the EOG timeseries to control for the possibility of eye movements driving decoding performance, and the impact of low‐level features was assessed by applying classifiers to image properties, specifically pixel intensity levels and the spatial envelope obtained using the GIST descriptor (Oliva, Hospital, & Ave, 2001). The latter consisted of 256 values for each image, obtained by applying Gabor filters at different orientations and positions to extract the average orientation energy. As such, the spatial envelope is a holistic representation of image properties, without extracting face‐specific features that can be expected to encode emotion and determine expression recognition.

## 3 RESULTS

### 3.1 Evoked responses to faces

**Figure 2:**
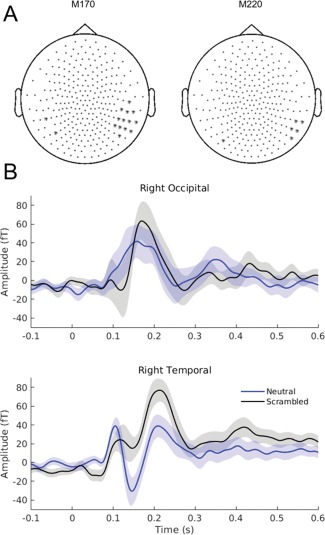
(a) Sensors exhibiting significant differences to faces compared to scrambled stimuli (marked with asterisks) at the M170 latency (left) and M220 latency (right; *p *<* *.01). (b) Timecourses of the evoked responses to neutral faces and scrambled stimuli from right occipital and temporal sensors averaged across subjects (±*SEM*) [Color figure can be viewed at http://wileyonlinelibrary.com]

When assessing the effect of emotional expression on event‐related fields, we found no modulation of any of the three ERF components (*F*(2,26) < 9.37, *p *>* *.061 across all three comparisons). Conversely, we found significant differences between responses to faces and scrambled faces at the M170 latency (*t*(1,13) > 5.43, *p *<* *.0078; maximum *t*(1,13) = 7.17, *p *=* *.0008) and at the M220 latency (*t*(1,13) > 5.38, *p *<* *.0099; maximum *t*(1,13) = 6.54, *p *=* *.0016; Figure 2). At the M100 latency, no differences survived correction for multiple comparisons (*t*(1,13) < 4.41, *p *>* *.04).

### 3.2 MVPA results: Decoding faces from scrambled stimuli

**Figure 3:**
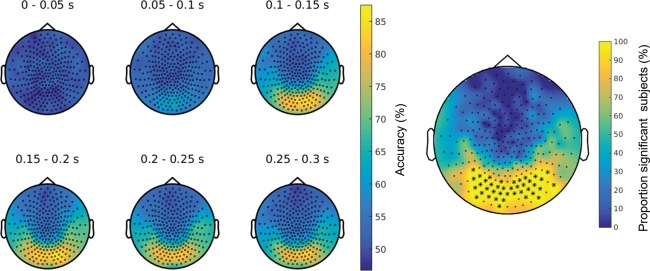
Searchlight MVPA analysis of differences in face/scrambled stimulus processing. The left‐hand panel summarizes time‐resolved decoding accuracy (averaged across subjects and 50 ms time windows). The right‐hand figure depicts the proportion of participants achieving above‐chance decoding at each sensor regardless of latency (sensors significant in all subjects and selected for further analysis are marked with asterisks) [Color figure can be viewed at http://wileyonlinelibrary.com]

A searchlight MVPA analysis was performed on the face versus scrambled decoding problem to identify sensors of interest for emotional expression classification. Faces were decoded above chance starting at ∼80 ms over occipito‐temporal sensors (Supporting Information Figure 2b). We identified a set of 40 occipito‐temporal sensors achieving above‐chance decoding performance in all participants at any time point after stimulus onset (Figure 3).

**Figure 4:**
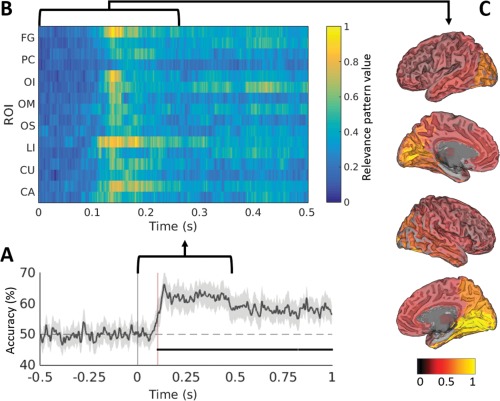
(a) Decoding accuracy for the face vs scrambled problem in source space with 95% CI and significant decoding time window (black horizontal line, starting at ∼100 ms). (b) Patterns derived from broadband source‐space decoding of faces and scrambled stimuli for 8 key ROIs for the 0–500 ms time window after stimulus onset. (c) Whole‐brain patterns averaged across the first 250 ms after stimulus onset and plotted on the semi‐inflated MNI template brain. Bilateral ROI labels: CA: calcarine cortex; CU: cuneus; LI: lingual gyrus; OS: occipital superior; OM: occipital medial; OI: occipital inferior; PC: precuneus; FG: fusiform gyrus [Color figure can be viewed at http://wileyonlinelibrary.com]

Source‐space face decoding showed a similarly early onset (∼100 ms), with slightly lower decoding accuracies. Relevance patterns based on classifier weights highlighted the visual cortex and fusiform gyrus between 100 and 200 ms post‐stimulus onset (coinciding with the M170 effects found in the ERF analysis; Figure 4).

### 3.3 MVPA results: Decoding emotional faces

#### 3.3.1 Sensor space decoding

**Figure 5:**
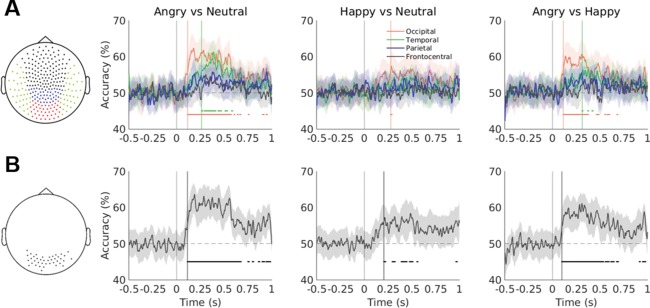
(a) Accuracy traces averaged across participants for each emotion classification problem and each of the four sensor sets (shown in the left‐hand plot). The vertical lines mark the stimulus onset and the shaded areas depict 95% bootstrapped CIs. The horizontal lines represent clusters of at least five significant timepoints (FDR‐corrected *p *<* *.05). Significant decoding onset is marked with vertical lines (at ∼100 ms for the angry vs. neutral/happy face decoding using occipital sensors). Accuracy traces were smoothed with a 10‐point moving average for visualization only. (b) As above for the sensor set based on the searchlight feature selection method (shown in the left‐hand plot) [Color figure can be viewed at http://wileyonlinelibrary.com]

When using anatomically defined sensor sets to define the feature space, MEG data from occipital sensors successfully discriminated angry and neutral faces (at 93 ms post‐stimulus onset), as well as angry and happy faces (at 113 ms post‐stimulus onset). The classification of happy and neutral faces was less consistent, showing only a weak effect (which reached significance for a brief time window at 278 ms). The temporal sensor set successfully decoded angry versus neutral faces starting at 262 ms. Other sensor sets did not achieve successful classification (Figure 5a). The maximum average accuracy across subjects was achieved in the occipital sensor set decoding of angry versus neutral faces (65.39%, bootstrap 95% CI [60.83%, 69.51%); Supporting Information Table S1).

Feature selection of sensors that successfully decoded faces versus scrambled stimuli marginally improved classification performance (Supporting Information Table S1) and led to above‐chance accuracy on all three pairwise classification problems, starting at ∼100 ms for angry faces and at ∼200 ms for happy and neutral faces (Figure 5b).

#### 3.3.2 Source space decoding

We used 84 peak virtual electrodes in AAL atlas‐based ROIs to perform whole‐brain decoding of emotional expression in source space. Angry faces were decodable from neutral faces at 155 ms and from happy faces at 300 ms, while happy and neutral faces were less successfully decoded, with a non‐significant peak at 363 ms.

Later onsets of significant effects in source space are likely to be due to the whole‐brain approach and the subsequently lower accuracies obtained in source space. Accuracy may have been decreased by the higher number of features and by our choice of one peak timecourse per ROI as input to the classification, which may have filtered out informative signal. However, as optimizing accuracy was not the main goal of this study, our method offers interpretability advantages, such as the ability to assess the relative roles of different ROIs without the confound of unequal ROI or feature vector sizes. Although feature selection could improve classification performance, we decided against optimizing accuracy in favor of deriving whole‐brain maps from classifier weights.

#### 3.3.3 Source‐space relevance patterns

**Figure 6:**
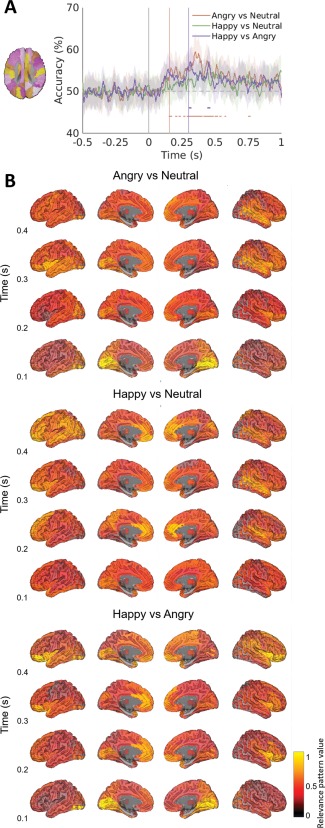
(a) Accuracy traces averaged across participants for each emotion classification problem in source space using the 84 AAL atlas‐based ROIs (shown in the left‐hand plot). (b) Broadband relevance patterns derived from classifier weights in source space for all three decoding problems, averaged across subjects and 100 ms time windows, baselined and normalized, mapped on the semi‐inflated MNI template brain for time windows between 100 and 500 ms post‐stimulus onset. Patterns visualized here are descriptive and represent each ROI in terms of its relative role in classification across subjects without statistical testing [Color figure can be viewed at http://wileyonlinelibrary.com]

**Figure 7:**
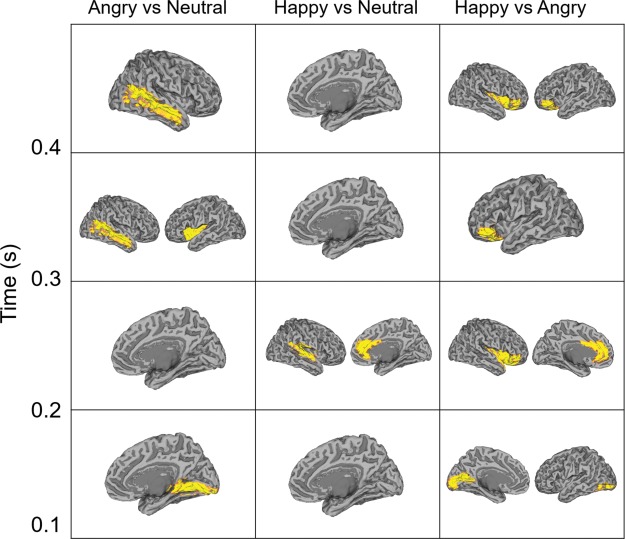
Results of permutation testing of relevance patterns shown in Figure 7 for each decoding problem and time window between 100 and 500 ms. Highlighted ROIs were assigned significant weights (*p *<* *.05 corrected) [Color figure can be viewed at http://wileyonlinelibrary.com]

To assess ROI contributions to source‐space decoding performance, classifier weights were converted into relevance patterns and then averaged across subjects and over time using 100 ms time windows. Relevance patterns attributed a key role to occipital regions within 200 ms of stimulus onset, with temporal and frontal regions contributing information at later stages (Figure 6). This was confirmed by permutation testing results, which highlighted the role of the right lingual gyrus in discriminating angry and neutral faces within 200 ms (Figure 7). Information in the left calcarine sulcus and inferior occipital gyrus (with a potential source in the occipital face area) appeared to differentiate angry and happy faces, while areas in the temporal, insular and inferior orbitofrontal cortices were involved at later stages in all three classification problems.

#### 3.3.4 Control analyses

For all three decoding problems, time‐resolved decoding performed on the EOG timeseries (using 25 time points from each of the two EOG channels as features) achieved a maximum accuracy no higher than 50.9% (bootstrapped 95% CIs [47.75%, 52.6%]). Classification performed on the entire EOG timeseries did not exceed 52.49% (CI [48.6%, 56.3%]). This suggests that decoding results were unlikely to be driven by eye movement patterns.

Binary classification between conditions based on raw image properties (intensity levels per pixel ranging between 0 and 1, mean 0.53, *SD* 0.16) was not significant, although suggestive for one decoding problem (happy vs. neutral: 33% accuracy, *p *=* *.9; angry vs. neutral: 60% accuracy, *p *=* *0.24; and angry vs. happy: 70% accuracy, *p *=* *.053, permutation testing).

Finally, we performed binary classification between pairs of emotional expression conditions, using the spatial envelope values calculated using the GIST descriptor for each image. Two of the decoding problems were successfully solved (happy vs. neutral: 82.6% accuracy, *p *=* *.0032, happy vs. angry: 78.7%, accuracy, *p *=* *.0062), while angry faces could not be decoded from neutral faces above chance (55.67% accuracy, *p *=* *.33). This suggests that in our stimulus set, visual properties distinguish happy faces from neutral and angry faces (unsurprisingly, given the consistency in happy expressions), while angry faces are not easily distinguishable from neutral faces. These results stand in contrast to results from MEG decoding (Supporting Information Figure S3), which follow an inverse pattern, with the highest accuracies obtained when decoding angry and neutral faces.

Together, the control analyses suggest that our MEG results cannot be readily explained by low‐level confounds in our stimulus set. The increase in accuracy when decoding angry faces from other expressions (∼100 ms), while likely to be based on low‐level information associated to emotional expression, is not easily explained by unrelated visual properties.

## 4 DISCUSSION

We used sensor‐space and source‐localized MEG data and data‐driven multivariate methods to explore the spatiotemporal dynamics of emotional face processing. We report three main findings based on our analyses. First, the emotional valence of faces (especially angry expressions) can be robustly decoded based on data from occipito‐temporal sensors, as well as whole‐brain source‐space data. Second, information related to emotional face category is available as early as 90 ms post‐stimulus onset, despite a lack of effects in trial‐averaged ERFs. Third, data‐driven relevance maps link different stages in expression perception to visual cortex areas (early stages) and higher‐level temporal and frontal cortices (later stages).

### 4.1 Early processing of facial expressions

Although we found no modulation of trial‐averaged ERF components by emotional expression, our ERF analysis revealed a face response over temporal sensors at the M170 and M220 latencies and no face‐specific M100 component, in line with previous studies using matched control stimuli and similar designs (Perry et al., 2014; Rossion, & Caharel, 2011). On the other hand, an early occipito‐temporal response to faces at M100 latencies was revealed in the MVPA analysis. Together, these results appear to point to different components in face processing—an early occipital effect not present in the trial‐averaged ERFs, and a later, mainly right‐lateralized temporal effect. Note that although the sensors contributing the most information to the MVPA analysis are different to the sensors identified in ERF analysis, the latter set of sensors do perform above chance when used in MVPA analysis in a majority of subjects (Supporting Information Figure S3); the increased heterogeneity can be explained by lower cross‐subject consistency at the sensor level of a late, higher‐level response.

Using MVPA, we were able to identify expression‐related information at early latencies in our sensor‐level MEG data. Expression (angry and neutral/happy faces) could be decoded at 93 and 113 ms respectively, only 10–30 ms later than faces were decoded from scrambled stimuli, and earlier than latencies reported by previous ERP studies (even by those showing emotional modulation of P1; e.g., Aguado et al., 2012). Such early latencies are consistent with neurophysiological investigations in primates: for example, multivariate analysis of LFP data in monkeys has shown early categorization of faces at 60–90 Cauchoix, Arslan, Fize, & Serre, 2012), while face‐selective cells in primate temporal cortex respond to faces or facial features at 80–100 ms (Perrett, Rolls, & Caan, 1982; Hasselmo, Rolls, & Baylis, 1989). Our results add to recent evidence of rapid visual categorization occurring during the early stages of ventral stream visual processing (Cauchoix et al., 2016; Clarke et al., 2013) and show that this extends beyond broad stimulus categories. Moreover, we reveal differences in patterns that can be detected in the absence of trial‐averaged ERF effects. Such differences, together with method heterogeneity, could explain previous mixed results in ERF studies, and speak to the sensitivity advantage of MVPA.

On the other hand, the lower performance and later onset of happy versus neutral face decoding suggests a categorization advantage inherent in angry expressions. Angry faces were decoded from both happy and neutral faces almost simultaneously, suggesting a bias related to threat and not to emotion in general. This points to a system preferentially responsive to threat, consistent with models placing conflict resolution at the core of social interaction (de Waal, 2000). We note that our whole‐brain, data‐driven analysis pipeline revealed this bias without entailing assumptions about the temporal or spatial location of an effect.

### 4.2 Spatial patterns of expression‐related information

We implemented an atlas‐based approach to our source‐space decoding analysis in order to improve the interpretability of the resulting maps and to facilitate cross‐modality comparisons (Hillebrand et al., 2012). This approach has been successfully applied to resting‐state MEG studies (e.g., Brookes et al., 2016) and, together with the selection of a peak source per ROI, allowed us to increase the computation speed of our whole‐brain decoding analysis, while at the same time reducing data dimensionality and allowing for direct comparison between ROIs. The relevance patterns in this study became stronger at time points corresponding to accuracy increases (starting at ∼100 ms), but we refrain from directly linking the two because we did not optimize accuracy in this study.

When decoding angry and neutral/happy faces, early differential processing was localized to the calcarine, lingual and inferior occipital ROIs, starting at ∼100 ms post‐stimulus onset (Figures 6 and 7). Other occipital ROIs showed a weaker contribution to decoding, with patterns later spanning a range of temporal and frontal areas. Early patterns differentiating neutral and happy faces were weaker (as confirmed by the lack of significant ROIs for this problem in the first 200 ms, and explained by the low decoding accuracy), but evolved similarly over time (Figure 7). Strong patterns in the early visual cortex and the occipital face area may be evidence of preferential threat processing based on coarse visual cues which are rapidly decoded and forwarded to higher‐level regions. Emotional modulation in the visual cortex has previously been reported (Fusar‐Poli et al., 2009; Herrmann et al., 2008; Padmala, & Pessoa, 2008), and the current results suggest that this effect occurs at the early stages of visual processing (within 200 ms in calcarine cortex and lingual gyrus).

The traditional model postulating different pathways for processing static facial features (such as identity) and changeable features (such as expression; Bruce & Young, 1986) has been challenged by mounting evidence of interaction between the two systems (Rivolta et al., 2016). Our results suggest that face‐responsive areas, including those thought to process identity, respond to emotional expression. The OFA/inferior occipital gyrus appears to be involved at an early stage, while the fusiform gyrus and the superior temporal ROIs (locations of the FFA and STS) are recruited at later time points. This is consistent with a hierarchical model based on feedforward processing of expression (Lohse et al., 2016; Wang et al., 2016). Later time windows are characterized by patterns in the insular, prefrontal and orbitofrontal cortices, previously associated with emotional processing especially at the later stages of integration and evaluation (Chikazoe, Lee, Kriegeskorte, & Anderson, 2014; Phan, Wager, Taylor, & Liberzon, 2002).

The timing of expression processing as evaluated with MEG MVPA can offer indirect evidence on the hierarchy of the modules involved. In the current study, the short latencies of emotional face discrimination in visual cortex can be interpreted as supporting a feedforward account of expression perception. Since we find the earliest differential effects in early visual cortex (within 100 ms), this appears to be somewhat inconsistent with the preferential relaying of expression information via the subcortical route to the amygdala (Pessoa et al., 2011). However, the current data are not incompatible with the possibility of a subcortical route with no preference to expression (Garvert et al., 2014; McFadyen et al., 2017).

Our results are in line with previous fMRI MVPA studies demonstrating above‐chance expression decoding in all face‐selective regions (Wegrzyn et al., 2015) and particularly in the FFA, STS, and amygdala, in the absence of univariate effects (Zhang et al., 2016a). Notably, the latter found that the STS could classify neutral and emotional faces above chance, whereas here we show an advantage for angry expressions. Further research is needed to elucidate the emergence of expression‐specific representations in the face‐selective network.

### 4.3 What does successful emotional face decoding tell us?

Naturalistic and high‐level stimuli, although appropriate for linking perception to cognitive processing, may give rise to ambiguities in interpretation. In this study, we attempted to strike a balance by matching low‐level characteristics across stimuli to the detriment of their naturalistic qualities. As emotional processing can encompass several distinct processes, we opted for a passive viewing paradigm that would eliminate task‐related or top‐down attention effects. Attentional effects would thus be expected to arise due to the stimuli themselves (increased salience due to emotional or threatening component) in a bottom‐up fashion compatible with our results. Without making claims as to the nature of the underlying processes, we argue that our design and results have relevance to real‐life perception of emotion in others.

Global low‐level matching of stimuli does not preclude the existence of local differences between images that are likely to play a part in early decoding. However, we note that the fact that angry faces are decoded more successfully than happy/neutral faces points to their relevance rather than to non‐specific decoding based on low‐level properties; for example, happy faces could be expected to be successfully decoded by a low‐level classifier due to their consistent smiles, as suggested by their successful decoding based on spatial envelope features. Furthermore, classification based on sensors that successfully discriminate between faces and scrambled stimuli adds to the evidence that our data do reflect face processing. It is likely that local low‐level properties play a part in decoding (especially in early time windows and low‐level visual areas); however, we view such properties as informative in the emergence of high‐level categories. Our results suggest that behaviorally relevant (threat‐related) low‐level cues are detected and relayed preferentially compared to benign emotional cues.

One limitation of our study is the fact that we could not perform cross‐exemplar decoding to test classifier generalization to a novel set of stimuli, as the occurrence of each exemplar was not recorded in our paradigm. Thus, there is a concern about the classifier potentially exploiting stimulus repetitions in order to successfully classify the two categories. However, as repetition numbers were balanced across conditions, we would expect this concern to affect all three decoding problems equally. As the control analyses do not point to the angry faces as more classifiable in terms of low‐level properties, we conclude that the successful decoding of angry faces from MEG data is consistent with their behavioral relevance and not with recognition of individual exemplars and stimulus properties. However, future studies could test the generalization of MEG responses to emotional faces to novel stimulus sets using cross‐classification in order to elucidate their mechanisms.

Furthermore, our choice of using stimulus repetitions to achieve robust responses to a limited stimulus set poses the concern of potential differences in repetition suppression effects. Such effects have been shown to covary with a number of factors, including time lag, task type, stimulus familiarity and valence (Morel et al., 2009). In particular, a stronger repetition suppression effect was shown for fearful faces than for neutral faces in both fMRI and MEG (Ishai, Pessoa, Bikle, & Ungerleider, 2004; Ishai, Bikle, & Ungerleider, 2006), although this effect was only present for target faces that were the object of a working memory task. On the other hand, repetition suppression was shown to be absent for happy faces and reduced for angry faces as compared with neutral faces in an fMRI study with an implicit paradigm (Suzuki et al., 2011). Such a pattern is inconsistent with a large contribution of repetition suppression effects to the current results. Furthermore, previous studies have shown differential repetition effects in evoked response potentials, while evoked responses in the current data revealed no differences between expressions. Due to stimulus labels not being recorded, we were unable to investigate this possibility further; however, future studies could test the robustness of expression‐specific neural responses to a larger stimulus set.

Although multivariate decoding is more sensitive to differences in neural responses than traditional methods, a necessary caveat of decoding‐based inference is that information relevant in classification may not be equally relevant in brain computation (Kriegeskorte, 2011). By restricting ourselves to a linear classifier, we ensure that the decoder focuses on information explicitly present in the neural activity, as the feature vector is only a linear transformation away from the neural data (King & Dehaenene, 2014; Ritchie & Carlson, 2016). However, it remains an open question whether the brain uses similar mechanisms in its own computations, whether non‐classifiable responses are otherwise represented in the brain, or indeed whether there is a causal link between such neural responses and perception or behavior.

On a similar note, patterns derived from classifier weights indicate the availability of decodable information, but it is difficult to assess the type of information used by the classifier or whether this same information is functionally relevant. However, our results are validated by existing models of emotional face processing, whereby large‐scale differences in spatial patterns over time may be elicited by different pathways involved in processing neutral and emotional/threat‐related and benign stimuli. On the other hand, the role played by individual ROIs in decoding can be interpreted as reflecting differences in neuronal population activity, as suggested by fMRI, MEG and electrophysiological investigations establishing correlations between face‐selective cell activity, the BOLD signal (Hasselmo et al., 1989; Tsao, Freiwald, Tootell, & Livingstone, 2006) and gamma oscillations (Muthukumaraswamy, & Singhgh, 2008; Perry, 2016; Perry et al., 2014). It is likely that different regions contribute different types of discriminating information and further study is needed to tease apart the underlying neural activity. While the overlap in areas between classification problems and the distributed nature of expression‐related information hint at the existence of a core system that efficiently identifies and relays emotional cues, the spatial resolution of our data is too coarse to make strong claims about the structure of this system.

The findings we discuss here extend beyond successful decoding of emotional stimuli to reveal a system optimized for rapid processing of emotional content in faces and particularly tuned to angry expressions. Decoding timecourses and relevance patterns indicate that affective information is rapidly relayed between early visual cortex and higher‐level areas involved in evaluation, suggesting that in our passive viewing paradigm, behavioral relevance impacts the processing speed of emotional expressions. Such a system is likely to confer an evolutionary advantage in terms of rapid processing of threat cues from other humans, consistent with models highlighting social processing skills as an essential asset in human evolution and the development of the human brain.

## Supporting information

Additional Supporting Information may be found online in the supporting information tab for this article.

Supporting Information
