# Supplementary material for: Spatiotemporal dynamics in human visual cortex rapidly encode the emotional content of faces
Source: Hum Brain Mapp. 2018 Jun 8;39(10):3993–4006. doi: 10.1002/hbm.24226 (PMC6175429; doi:10.1002/hbm.24226)
Supplement: Supplementary file 1 — Supporting Information [file HBM-39-3993-s001.docx]

## Supporting Information


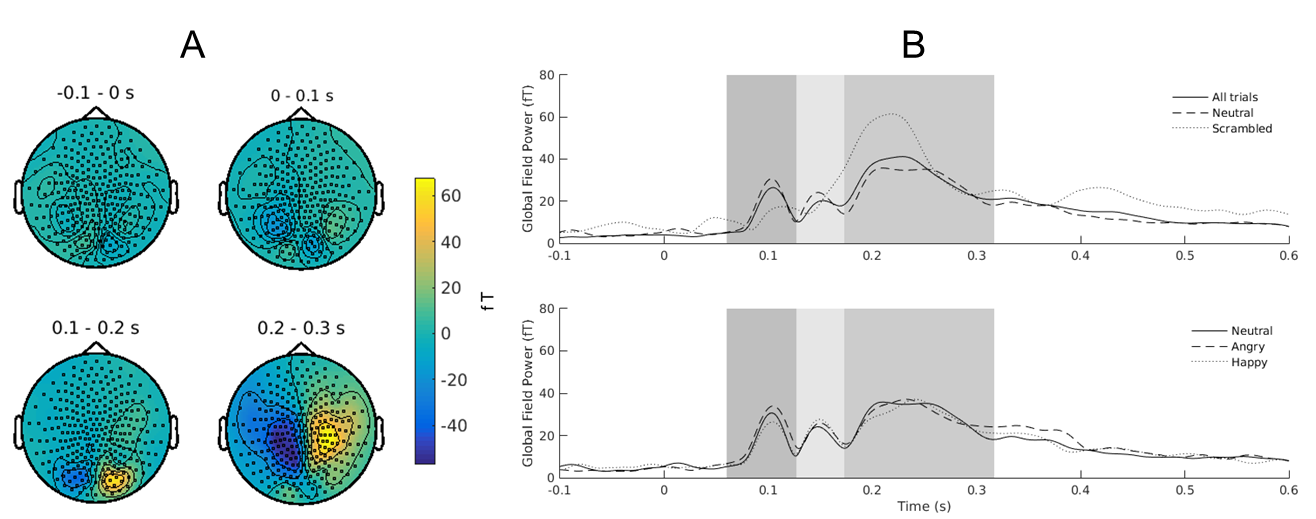


**Supplementary Figure 1. A.** Topographical distribution of the grand average ERF amplitudes from all axial gradiometers across the three face conditions. **B.** Global field power of the grand average ERF across all trials and for each condition. Shaded areas show windows of interest in the ERF analysis.


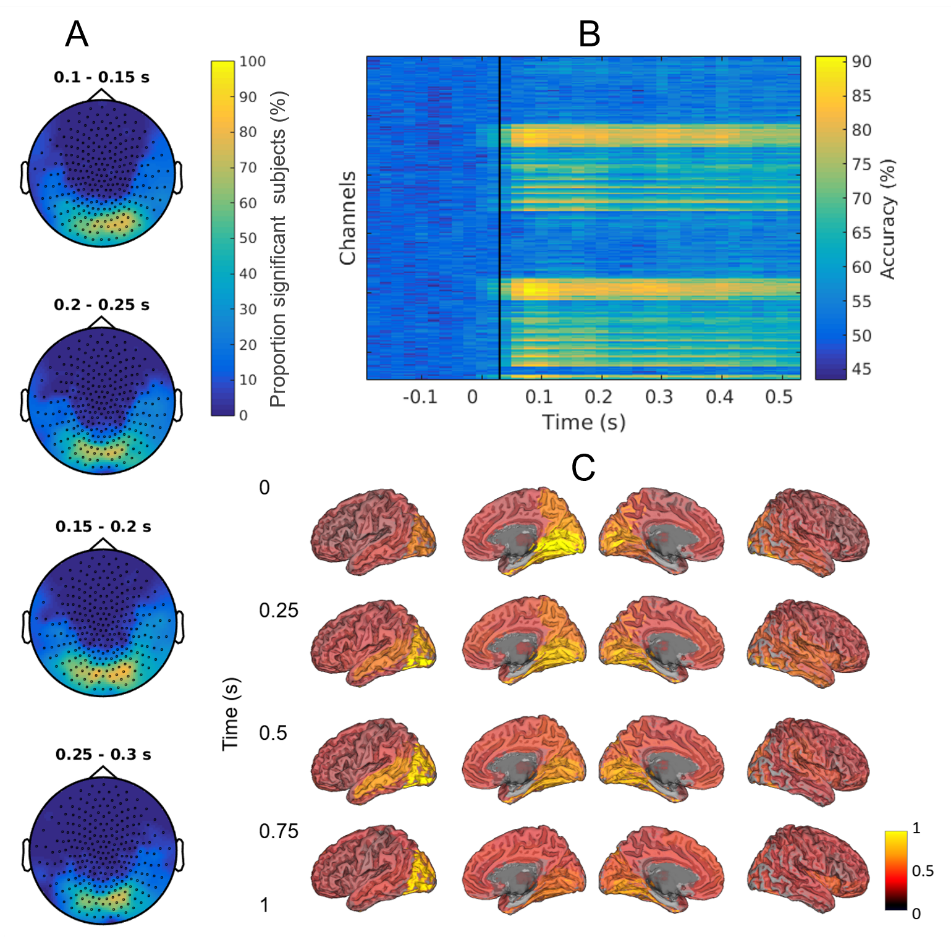


**Supplementary Figure 2. A.** Proportion of subjects achieving significant face vs scrambled stimulus decoding over time in the searchlight MVPA analysis (numbers are averaged over 50 ms time windows for visualization). **B.** Accuracy over time (without averaging) in the same analysis. The black horizontal line marks the onset of above-chance decoding (80-110 ms). **C.** Source-space relevance patterns for the face vs scrambled decoding problem, averaged across subjects and 250 ms time windows.

**Supplementary Table 1. Emotional expression decoding results**

|  |  | Occipital | Temporal | Parietal | Frontocentral | Selected sensors | Source space |  |
| --- | --- | --- | --- | --- | --- | --- | --- | --- |
| Angry vs Neutral | Max accuracy | 65.39% | 63.68% | 58.62% | 57.52% | 65.96% | 61.13% | |
|  | Bootstrap  95% CI | 60.83%, 69.51% | 58.91%, 68.68% | 56.20%, 61.59% | 52.28%, 60.23% | 62.03%,  69.11% | 57.41%, 64.77% | |
|  | Max F1 score | 0.653 | 0.636 | 0.585 | 0.573 | 0.659 | 0.611 | |
|  | Peak time point | 267 ms | 388 ms | 947 ms | 618 ms | 185 ms | 376 ms | |
|  | Decoding onset | 93 ms | 262 ms | N/A | N/A | 113 ms | 155 ms | |
|  |  |  |  |  |  |  |  | |
| Happy vs Neutral | Max accuracy | 59.97% | 58.23% | 58.14% | 57.30% | 60.65% | 58.98% | |
|  | Bootstrap  95% CI | 55.11%, 65.27% | 55.37%, 61.01% | 54.36%, 63.28% | 53.32%, 61.56% | 57.05%,  65.22% | 56.31%, 61.12% | |
|  | Max F1 score | 0.599 | 0.581 | 0.58 | 0.572 | 0.605 | 0.589 | |
|  | Peak time point | 485 ms | 315 ms | 673 ms | 637 ms | 481 ms | 363 ms | |
|  | Decoding onset | 278 ms | N/A | N/A | N/A | 205 ms | N/A | |
|  |  |  |  |  |  |  |  | |
| Happy vs Angry | Max accuracy | 62.83% | 62.29% | 57.97% | 57.02% | 64.03% | 60.93% | |
|  | Bootstrap  95% CI | 59.70%, 66.88% | 57.18%, 65.60% | 54.43%, 63.06% | 53.21 %, 60.55% | 59.32%,  69.87% | 57.29%, 64.91% | |
|  | Max F1 score | 0.628 | 0.621 | 0.578 | 0.568 | 0.639 | 0.609 | |
|  | Peak time point | 332 ms | 468 ms | 465 ms | 403 ms | 313 ms | 455 ms | |
|  | Decoding onset | 113 ms | N/A | N/A | N/A | 98 ms | 301 ms | |

**
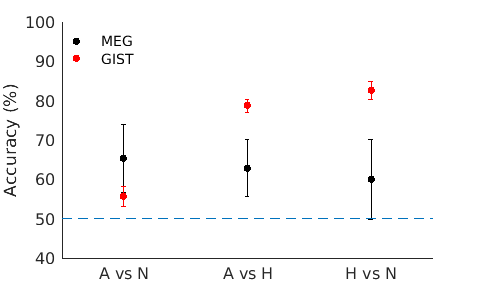
**

**Supplementary Figure 3.** Decoding results obtained using the MEG occipital sensor set at peak time point across subjects, and the spatial envelope calculated using the GIST descriptor (error bars: bootstrap 95% CI based on classification across subjects/cross-validation iterations), when decoding emotional expression (A: angry; H: happy; N: neutral). The blue dashed line marks the theoretical chance level (although note that the angry vs neutral GIST-based classification does not exceed the empirically estimated chance level).

**Supplementary analysis 1: Feature selection**

Emotional expression classification was performed using: (1) the 15 sensors found to exhibit significant differences in ERFs between faces and scrambled stimuli in any of the three time windows tested; and (2) a combined set of 55 sensors identified through the MVPA and ERF-based feature selection methods (see Methods).

| Method |  | Angry vs Neutral | Happy vs Neutral | Happy vs Angry |
| --- | --- | --- | --- | --- |
| ERF | Maximum accuracy | 61.68% | 57.56% | 59.75% |
|  | Bootstrap 95% CI | 58.68%, 64.93% | 52.87%, 62.21% | 56.27%, 61.67% |
|  | Maximum F1 score | 0.616 | 0.574 | 0.596 |
|  | Peak time point | 390 ms | 771 ms | 313 ms |
|  | Decoding onset | 175 ms | N/A | N/A |
| Joint | Maximum accuracy | 65.59% | 61.7% | 63.4% |
|  | Bootstrap 95% CI | 60.97%, 69.5% | 56.34%, 66.31% | 60.56%, 68.34% |
|  | Maximum F1 score | 0.655 | 0.616 | 0.633 |
|  | Peak time point | 181 ms | 438 ms | 352 ms |
|  | Decoding onset | 116 ms | 203 ms | 110 ms |

**Supplementary Table 2.** Results of emotional face decoding using both feature selection methods described above. Results are comparable to those observed using the MVPA-based sensor set and do not bring an improvement in decoding performance.


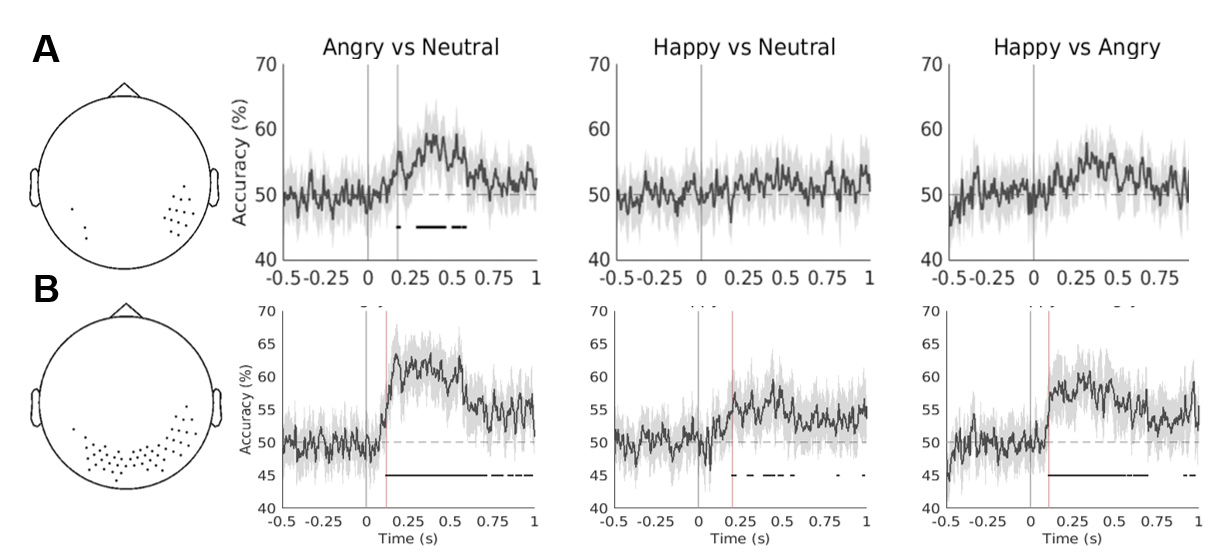


**Supplementary** **Figure 4.** Time-resolved decoding performance for each of the emotional face problems using **A.** the ERF-based sensor set; **B.** the joint sensor set.

**Supplementary analysis 2: Source-localized ROI evoked responses**

Both ERF and MVPA analyses at the sensor levels pose concerns in terms of alignment between subjects, with sensors exhibiting significant effects across subjects not necessarily reflecting comparable brain activity due to differences in head position and anatomy. Source localization can alleviate these concerns, by aligning individual anatomy to a template and computing the sources contributing to the sensor-level data.

Thus, to assess whether the MVPA effect found at the source level was also present in univariate responses when eliminating the issues associated with sensor-level analyses, we calculated evoked responses (trial averages) for the peak sources in each of the 84 ROIs used in the MVPA source-space analysis (see Methods).

For the face vs scrambled analysis, the figure below shows the grand average evoked response (across subjects and ROIs). Statistical analysis on the ROI-averaged response using the three time windows described in the manuscript (M100, M170 and M220; lines 145-146) revealed a significant difference only in the M170 window (*P*=0.0012, t(13)= -4.89; paired T-test and randomization testing using 5,000 iterations). Tests performed at each ROI were inconclusive (*P*>0.09, t(13)<4.1). We note here that the selection of one source per ROI and the number of comparisons performed are likely to be the cause of these results.


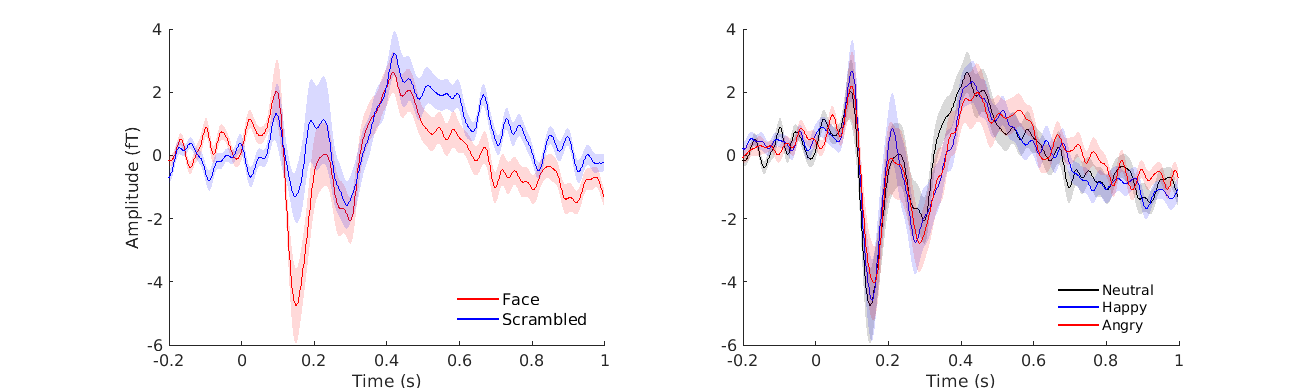


**Supplementary** **Figure 5.** Grand average evoked responses (±SEM) over 84 ROIs and all 14 subjects.

For the emotional expression analysis, we performed repeated-measures ANOVA and randomization testing (5,000 iterations) on both ROI-averaged data and at each ROI separately using the same three time windows of interest. Neither of these approaches revealed significant results (*P*>0.22, F(2,26)<1.58, and *P*>0.25, F(2,26)<6.5 respectively). We note that apart from exploiting information in multivariate patterns, our MVPA framework also alleviates the multiple comparison problem while still assessing whole-brain effects, thus achieving better sensitivity to statistical differences. Moreover, information is inevitably lost in trial averaging; the sensor and source space MVPA analyses point to an early stage of visual processing in which behaviourally relevant emotional cues are differentiated, but which is not captured by the ERFs.
